# Sociocultural Influences on the Feeling of Loneliness of Family Caregivers of People with Dementia: The Role of Kinship

**DOI:** 10.3390/ijerph18094700

**Published:** 2021-04-28

**Authors:** Cristina Huertas-Domingo, María Márquez-González, Isabel Cabrera, Samara Barrera-Caballero, María del Sequeros Pedroso-Chaparro, Rosa Romero-Moreno, Andrés Losada-Baltar

**Affiliations:** 1 Departamento de Psicología, Universidad Rey Juan Carlos, 28922 Alcorcón, Madrid, Spain; cristina.huertasd18@gmail.com (C.H.-D.); samara.barrera@urjc.es (S.B.-C.); maria.pedroso@urjc.es (M.d.S.P.-C.); rosa.romero@urjc.es (R.R.-M.); 2 Facultad de Psicología, Universidad Autónoma de Madrid, 28049 Madrid, Spain; maria.marquez@uam.es (M.M.-G.); i.cabrera@uam.es (I.C.)

**Keywords:** caregivers, dementia, familism, dysfunctional thoughts, kinship, loneliness

## Abstract

The extent to which familism, dysfunctional thoughts, and coping variables contribute to explaining feelings of loneliness in caregivers, controlling for kinship, is analyzed. Participants were 273 family caregivers of people with dementia. Sociodemographic variables, familism, dysfunctional thoughts, coping strategies for requesting and receiving help, perceived social support, and leisure activities were assessed. The fit of a theoretical model for explaining the effect of cultural and psychological variables on feelings of loneliness in each kinship group was tested. No significant differences in the distribution of loneliness by kinship were found. Higher levels of familism are associated with more dysfunctional thoughts, that are linked to more maladaptive strategies for coping with caring (e.g., less social support and fewer leisure activities). This in turn is associated with higher scores in the feeling of loneliness. The model bore particular relevance to the group of daughters, husbands, and sons, yet not in the case of wives. Sociocultural and coping factors associated with the caring process seem to play an important role in explaining feelings of loneliness in caregivers. Sociocultural factors associated with the care process seem to play an important role in explaining feelings of loneliness in caregivers.

## 1. Introduction

The progressive aging of the population due to increasing life expectancy is associated with a significant increase in the risk of dementia in Western countries [1] and in the number of people needing care [2]. The care of relatives that have dementia is commonly provided by families and, more specifically, by one person who adopts the role of primary caregiver, usually a woman [3]. Family care of a relative with dementia is associated with significant negative physical and psychological consequences for the caregiver, among which are the presence of anxious and depressive symptoms [4] and loneliness. Loneliness has scarcely been studied in caregiving contexts, despite the presence of studies suggesting its great prevalence among caregivers [5,6,7]. Such negative consequences associated with caregiving appear to occur to a greater extent among women [8], with studies suggesting that caregiver daughters in particular are at greater risk of suffering emotional problems [9].

Different theoretical models have explained the presence of emotional distress in caregivers, with the stress and coping model proposed by Lazarus and Folkman [10] salient among them. According to this model, apart from the sources of the stress itself, the consequences of caregiving-related stress depend on how the person interacts with the situation based on their evaluation of it and the personal and social resources available to them. Based on the Lazarus and Folkman [11] model, theoretical models adapted to caregiving have been developed, such as the sociocultural stress and coping model, which has significant empirical support [11]. This model offers a conceptual framework of caregiver stress and the different coping processes as influenced by cultural factors [11]. One of the cultural values which has received the most attention in the study of care is familism [11,12]. This variable reflects a cultural value related to solidarity between the different members of the family associated with strong feelings of loyalty and dedication to one another [13]. Thus, while factors such as family solidarity or affection may have a role to play in family caregiving, such care also involves the fulfillment of a duty, so that caring for the family member is combined with obligation. The provision of care in a context where the perception of family obligation is strong is associated with a significant negative impact resulting in emotional distress for caregivers [12].

The effect of cultural variables in caregiving seems to be greater on women due to the traditional attitudes associating care with the female role, so that this activity continues to fall more heavily on them [14]. This distribution of traditional gender roles may be at the root of why women, in contrast to men, become involved to a greater extent in caregiving tasks and therefore deal with more demands for care and spend more time on it [8]. Furthermore, the fact that women habitually have to play multiple roles (worker, wife, daughter, mother, etc.) makes them more likely to experience the adverse consequences of caregiving [15].

These considerations are in line with those posited by the cognitive model adapted to caregiving [16]. This model hypothesizes that culture clearly influences how caregiving is seen and handled (e.g., “Only the closest person to the frail/sick older adult knows how to truly take care of him or her”; “It is selfish for a caregiver to dedicate time to himself/herself when a relative is frail/sick and needs care”). Such thoughts can be linked to behaviors which may have negative physical and psychological consequences for health, such as not asking for help or not engaging in leisure activities. Failure to ask for help can reduce the possibilities of maintaining social relationships (support network), leisure, motivation, and energy, thereby changing the structure of one’s social network, which can result in isolation [17] and loneliness [7]. Although scarce, the available literature on loneliness in caregivers seems to suggest that female caregivers report higher levels of loneliness [6] and, according to the results obtained by Stefani, Seidmann, Pano, Acrich, and Pupko [18], daughters show greater loneliness than wives.

**Figure 1:**
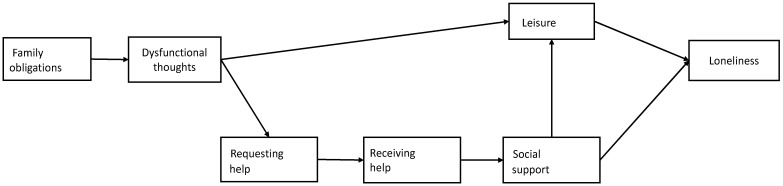
The sociocultural stress and coping model tested.

Taking into account all of the above, and based on the sociocultural [11] and cognitive [16] models of care-related stress and coping, the present study therefore had the following objectives: (a) to assess the degree to which perceived loneliness is present in caregiving and analyze potential kinship-based differences in the degree of perceived loneliness; (b) to evaluate the fit of a theoretical model analyzing the degree to which, by kinship, cultural values are associated with dysfunctional thoughts and the maladaptive coping strategies of requesting and receiving less help, as well as having less social support and fewer leisure activities, and (c) to analyze how these relationships influence the perception of loneliness in family caregivers of people with dementia (see Figure 1).

Along these lines, we expected (1) to find a significant percentage of caregivers, especially women and in particular daughters, reporting feelings of loneliness; (2) that a greater presence of the cultural value of familism would be associated with more dysfunctional thoughts about caregiving, which would be linked to the existence of more maladaptive coping strategies for caregiving, that is, requesting and receiving less help, which would in turn lead to a lower predisposition to social support and a lower frequency of leisure activities; and (3) that a greater implementation of such maladaptive coping strategies would be reflected in higher scores in the feeling of loneliness among caregivers. Given the influence of gender roles, the aforementioned consequences were expected to be especially significant in caregiver daughters as they are subject to greater role conflict.

## 2. Method

### 2.1. Participants

The study sample comprised 273 people (women = 68.9%) aged between 28 and 88, all of them caregivers of relatives with dementia residing in the Community of Madrid (Spain). Participants were divided into four kinship-based groups: husbands (n = 56; 20.5%), wives (n = 67; 26.48%), daughters (n = 117; 42.85%), and sons (n = 33; 12.08%). The sociodemographic characteristics of participants by kinship are shown in Table 1.

Participants were recruited through health and social services centers, which facilitated initial contact to confirm criteria for inclusion in the study were met (identifying as the main caregiver of the sick relative, dedicating at least one hour a day to caregiving and having done so for at least the previous 3 months, and being aged over 18). All subjects participated voluntarily and were individually interviewed face-to-face. The study was approved by the Ethics Committee of the Rey Juan Carlos University, and prior to the assessment, informed consent, guaranteeing data confidentiality among other issues, was obtained from each individual.

### 2.2. Measures

Drawing upon the sociocultural stress and coping model, the following variables were measured.

Sociodemographic variables: age, gender, kinship to the person being cared for, daily hours caring, paid work outside the home, living with children at home, and caring for other relatives.

Care-recipient functional capacity. This was measured through the Barthel Index [19], which showed internal consistency (Cronbach’s alpha) of 0.91 in this study.

Frequency of behavioral problems associated with dementia. This was measured through the Revised Memory and Behavior Problems Checklist (RMBPC) [20]. It includes 24 items (e.g., “Asking the same question over and over”) which assess the frequency of behavioral problems scored on a 5-point Likert scale ranging from 0 (“Never”) to 4 (“Every day”). The internal consistency (Cronbach’s alpha) in this study was 0.78.

Familism: family obligations. This was assessed through the family obligations subscale of the Revised Familism Scale (RFS) [21]. It consists of 5 items (e.g., “One should make great sacrifices in order to guarantee a good education for his/her children”) with a response range from 0 (“Totally disagree”) to 4 (“Totally agree”). The subscale’s internal consistency (Cronbach’s alpha) for this study was 0.74.

Dysfunctional thoughts. The Dysfunctional Thoughts Questionnaire (CPD) [22] was used to evaluate thoughts, beliefs, and attitudes that hinder adaptive coping related to caregiving. It consists of 16 items (e.g., “Only the person closest to the frail/sick older adult knows how to truly take care of him or her”) with a response range from 0 (“Totally disagree”) to 4 (“Totally agree”). Internal consistency (Cronbach’s alpha) in the present study was 0.91.

Requesting and receiving help. These were assessed by administering two items developed ad hoc for this study (“Do you ask your relatives for help with caring for your sick relative?” and “Do you receive help?”). Both items have a 4-option Likert-type response format ranging from 1 (“Never”) to 4 (“Always”).

Social support. The Psychosocial Support Questionnaire (PSQ) [23] was used to assess the perception of social support, using 6 items (e.g., “My friends and/or relatives pay me visits at home”) in a Likert-type response format with a response range from 0 (“Never”) to 3 (“Very often”). Cronbach’s alpha in the present study was 0.76.

Frequency of leisure activities. This was measured using the adapted Leisure Time Satisfaction scale (LTS) [24]. Its 6 items (e.g., “Quiet time by yourself”, “Taking part in hobbies”) measure the extent to which caregivers had participated in leisure activities. Responses are rated on a 5-point Likert-type scale from 0 (“Not at all”) to 4 (“A lot”). Cronbach’s alpha for this scale in the present study was 0.71.

Feeling of loneliness. Item 14 (“I felt lonely”) of the CES-D scale [25] with a response range from 0 (“Rarely or never”) to 3 (“All the time”) was used to measure how lonely people felt. This item has been used as an indicator of loneliness in previous studies (e.g., [26]) and is the same or similar to that included in different instruments to assess loneliness, such as the OARS scale [27].

### 2.3. Data Analysis

Statistical Package for the Social Sciences (SPSS statistics 22) (IBM, Chicago, IL, USA) and AMOS 6.0 software (IBM, Chicago, IL, USA) were used to carry out the analyses for this study. Considering the small sample size of the group of sons, the associations that are significant at *p* < 0.10 were highlighted for this group. A *p* value of 0.05 is considered for the rest of the results. First, descriptive analyses, comparison of means, and independence tests were carried out between the sociodemographic variables assessed and the perception of loneliness. Second, correlation analyses were performed between the different variables for each kinship group (presented as Supplementary Materials). Finally, and following the sociocultural model of stress and coping [11] and the cognitive model adapted to caregiving [12,16], we assessed the fit of the data to the model presented in Figure 1 through path analysis. The cultural variable of family obligations is taken first, followed by dysfunctional thoughts and caregiver resources (asking for help, receiving help, social support and engagement in leisure activities) and, finally, the feeling of loneliness. The associations found in the model for each of the kinship groups were analyzed. The following indices were used to assess the fit of the data from the model: chi-square, incremental fit index (IFI), comparative fit index (CFI), and the root mean square error of approximation (RMSEA).

## 3. Results

### 3.1. Sample Characteristics by Kinship

Table 1 shows the descriptive characteristics for each of the variables studied by kinship group. As can be seen, significant differences were found between these groups for almost all the variables assessed, with the exception of receiving help, leisure and loneliness. The percentage of caregivers who reported feeling lonely occasionally or most or all of the time ranged from 25.6% (daughters) to 30.5 (wives).

The data show that more daughters and sons work outside the home than husbands and wives. Daughters and sons also report a smaller number of daily hours devoted to caregiving compared to wives and husbands. Daughters as a group are those who most report living with children and who seem to be caring the most for other family members. Finally, daughters report caring for people with more problem behaviors than the other kinship groups, and they also care for more people with lower functional capacity than wives and husbands.

In turn, it is also daughters who present differences in family obligations compared to the other groups, where significantly lower scores in this variable are found. Regarding dysfunctional thoughts, results show that husbands and wives present more dysfunctional thoughts about caregiving than sons and daughters. In relation to asking for help, the data suggest that sons as a group ask for the most, while wives are the least likely to implement this caregiving coping strategy. However, in terms of social support, significant differences are seen in this variable between sons and the other groups, with sons in this case scoring lower on social support received. The associations between the assessed variables (correlations) are presented in the Supplementary Materials.

### 3.2. Model Fit and Associations by Kinship Group

**Figure 2:**
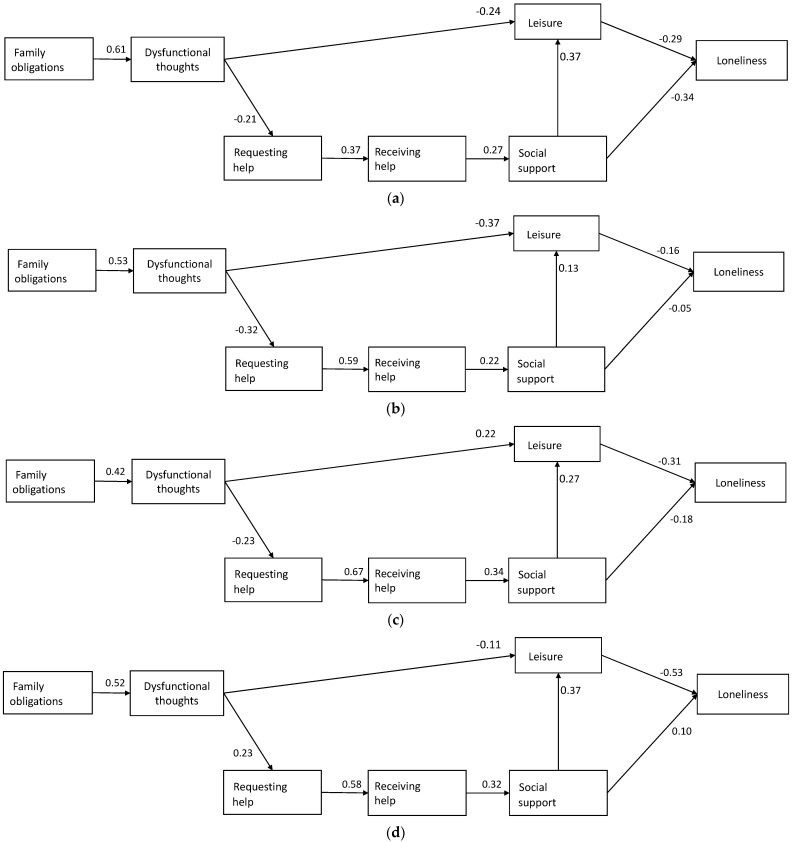
Standardized regression weights for (**a**) daughters, (**b**) wives, (**c**) husbands, and (**d**) sons.

The overall model fitted the data very well (χ^2^ = 67.31; *p* = 0.075; χ^2^/df = 1.29; IFI = 0.956; CFI = 0.946; RMSEA = 0.033). Table 2 shows the unstandardized estimates for each of the kinship groups assessed. The standardized regression weights for each kinship group are shown in Figure 2.

As can be seen in Table 2 and Figure 2, all the associations between the variables shown in the model are significant in the group of daughters. In this group, the perception of family obligation is associated with more dysfunctional thoughts, which in turn are associated with asking for and receiving less help. Receiving less help is associated with less perceived social support. Less social support and more dysfunctional thoughts are associated with less engagement in leisure activities. Finally, less engagement in leisure activities and less social support are associated with a greater perception of loneliness. The relationship between these variables explains 27% of the variance of loneliness in daughters.

In the case of wives, results are very similar to those of daughters in terms of the significant relationship between the variables family obligations, dysfunctional thoughts, asking for help, receiving help, and the relationship between receiving help and social support (*p* < 0.10) and dysfunctional thoughts and less leisure. However, contrary to our hypotheses, no significant relationship was found in wives between social support and leisure, and feelings of loneliness (Table 2). The model tested explains only 3% of the variance of loneliness in wives.

Regarding husbands, practically all the associations observed in daughters and stated in the model are also present (some with a *p* < 0.10), with the exception of the relationship between social support and loneliness, which seems to be mediated by leisure. The model explains 16% of the feeling of loneliness in husbands.

Finally, in the case of caregiver sons, the data obtained are somewhat different from the other groups. In this case, despite finding the significant association observed in all groups between family obligations and dysfunctional thoughts, dysfunctional thoughts were not significantly associated in sons with asking for help or with leisure. As with husbands, the pathway that seems to influence the feeling of loneliness most is that by requesting less help, less help is received, less social support is perceived, there is less engagement in leisure activities, and this influences loneliness. The percentage of variance explained in sons is 25%.

## 4. Discussion

The main objective of this study was to analyze the degree to which perceived loneliness is present in caregiving and whether there are differences in perceived loneliness based on kinship. The results obtained are consistent with other studies in that they reflect perceived loneliness being present in a significant percentage of caregivers [18]. However, although it was expected that daughters would present the highest levels of loneliness due to their being subjected to greater stress and role conflict, the results of this study suggest that there are no kinship-based differences in the degree of perceived loneliness. Therefore, the first hypothesis posited in this study is partially fulfilled, since the data do suggest a high presence of perceived loneliness among caregivers, but not greater loneliness in the group of daughter caregivers compared to other relatives. These results may be due to the fact that a large percentage of daughters have paid work outside the home, a rare occurrence among older groups (husbands and wives). Work is not only a way of earning a salary or gaining social prestige, but it also allows greater participation in social life and can act as a protective factor against the feeling of loneliness [28]. On the other hand, results also show daughters to be the group which most lives with children, most takes care of other family members, and more frequently deals with the disruptive behaviors and functional deterioration of the person in their care. These data seem to confirm that daughters are subjected to more sources of stress [8], which can lead to role overload and higher levels of stress, and which in turn may favor or be related to less awareness of their levels of loneliness.

With regard to the second objective, assessing the fit to the data of a stress and coping model based on sociocultural [11] and cognitive [16] models, the results appear to confirm the proposed hypotheses, especially in the group of daughters. In relation to daughters, these hypotheses seem to be supported by the data since a greater perception of family obligations is linked to a greater sense of loneliness, given that the influence of such obligations on dysfunctional thoughts regarding caregiving favors the implementation of maladaptive strategies (failure to ask for and receive help) and with it less social support and leisure. In this sense, the data also support the cognitive model adapted to caregiving [16]. In particular, our data support the pathological way through which culture has an influence on the development of maladaptive thoughts in caregivers (e.g., “I should not ask for help, this is something that should be resolved in the family”), which have negative consequences in terms of behavior (no help seeking, no leisure time) and affective consequences (loneliness).

For the groups of male caregivers (husbands and sons), the results obtained are generally similar to those observed in daughters, although some differences were noted. In both husbands and sons, for example, the level of perceived social support does not appear to be of particular relevance in explaining their perception of loneliness. The results suggest that in male groups (husbands and sons) the relationship between social support and loneliness could be indirectly mediated through leisure. Different studies suggest that men, despite requesting help in a similar way to women, receive more social support (e.g., [29]). In the case of caregiver sons, this is the group in our study (data not shown) with most singles, where the majority do not have children and practically all of them (all but 3) care for their mother. It is possible that they took on their role as caregivers because no other possible sources of potential caregivers existed in their environment and that the main route of escape from their loneliness was therefore perceived to be through leisure. Future studies should address these issues in more depth.

The most unexpected results were found in caregiver wives, for whom the proposed model explains only 3% of their perceived feeling of loneliness. Although the data obtained suggest that a significant percentage of caregiving wives report loneliness, the variables measured do not seem to contribute to an explanation of this issue in the group in the same way they do in the other kinship groups. It is possible that variables which were not assessed in this study may play a fundamental role in the explanation of loneliness among wives, such as, for example, the loss of a relationship of intimacy and trust with their partner, as well as maladaptive strategies for the regulation of emotions or coping, such as rumination or escape-avoidance coping strategies. Thus, for example, Vikström, Josephsson, Stigsdotter-Neely, and Nygard [30] reported that many couples attributed their perception of loneliness to the loss of an intimate relationship, since their partner, their confidant, provided them with great emotional support. Regarding rumination, the study by Robinson-Whelen Tada, MacCallum, McGuire, and Kiecolt-Glaser [31], carried out exclusively with spousal caregivers, mostly women, the authors concluded that the variable that contributed most to the emotional distress of the caregivers was intrusive and avoidant thoughts about caregiving. Robinson-Whelen et al. [31] also stated that social support received during the caregiving years was not so much related to negative outcomes as social support after the loss of the spouse. Social support during caregiving may not be so relevant for female spouse caregivers because caring for the husband is coherent with the gender-role script. Finally, as reviewed by Yee and Schulz [32], compared with men, women reported using more escape-avoidance coping strategies, suggesting the usefulness of training them in seeking-support strategies (e.g., assertive communication).

Therefore, although loneliness seems to be a pervasive problem among caregivers, there appear to be different explanations for it depending on the kinship of the caregiver with the person in their care. In terms of the influence of familism and the dimension of family obligations in particular, the data indicate a lower presence of familism (family obligations) in the group of daughters and less perceived social support in the group of sons. In relation to familism in daughters, the reduced presence of this variable may be explained by the conflict that this value can trigger in them with respect to the situation in which they find themselves. Daughters may be aware of how the sense of family obligation falls mainly upon them and, therefore, they question it more than other groups since the consequences are much greater for them [32]. Montgomery [33] argues that women, especially those who work outside the home, have greater difficulty in combining work and family, and experience greater social limitations than men, something that contributes to their sense of distress. Regarding perceived social support, the fact that sons show lower levels of this may be explained by taking into account that when sons take on the role of caregiver in a society where the traditional family values prevail and caring seems to be fundamentally associated with the daughters, it may be because there is no one else who can do it, indicating that they are more alone in the caregiving situation. In fact, authors such as Hanlon [34] identify two types of male caregivers: those who have no other option, that is, who are forced into caregiving, with the result that their participation in the labor market decreases, and those who have nothing to lose. Therefore, although the literature supports the argument that men receive more help with caregiving than women, this may not be enough to generate high levels of perceived social support since they are usually in a vulnerable situation, without their own family or children and without a job or career, and caring for their mother because her spouse can no longer do so.

The present investigation has a series of limitations. First, the design was cross-sectional in nature, and longitudinal studies are necessary to confirm or rule out the relationships found. Second, participation in the study was voluntary, so the general population of caregivers may not be properly represented. Furthermore, as noted above, variables which may be of particular relevance to understanding the feeling of loneliness in caregivers were not included in the study. Future research with larger samples may allow a more detailed inquiry into the relationships studied.

Despite such limitations, the present study provides interesting information on an area that generates significant distress among caregivers: loneliness. First, the data confirm that a significant percentage of caregivers perceives loneliness. Second, data from the present study provide additional support to theoretical models of stress and coping adapted to caregiving which highlight the importance of sociocultural aspects in the explanation of caregiver distress [11,12], in this case aimed at explaining the loneliness of caregivers. They also support the cognitive model adapted to caregiving [16] since in the case of daughters in particular, they suggest that cultural variables play an important role in the perception of loneliness. These cultural variables include familism (family obligations) and the dysfunctional thoughts of caregivers, which give rise to maladaptive coping with caring, thereby reducing the chances of obtaining help, social support and engagement in leisure activities. Caregiving daughters seem to be particularly vulnerable to this process since the variables measured explain a large percentage of the loneliness they feel.

Finally, the data from this study confirm that loneliness is a pervasive problem in caregiving and suggest an approach to understanding the issue which can be useful in developing interventions. Such interventions could help caregivers towards a more flexible view of the cultural impositions associated with family obligations in caregiving and maladaptive thinking styles, perhaps through culturally sensitive interventions [35].

## Figures and Tables

**Table 1:** Kinship differences in the assessed variables.

Variables	0. Daughters		1. Wives		2. Husbands		3. Sons		F/χ^2^ (sig)	Diff.
	(n = 117)		(n = 67)		(n = 56)		(n = 33)			
	Mean	SD	Mean	SD	Mean	SD	Mean	SD		
Age	54.07	8.22	72.34	8.06	75.21	6.64	52.87	9.94	98.49 (0.00)	1 & 2 > 0 & 3
Loneliness never (%)	43.5		46.2		57		48.5		10.75 (0.29)	n.s.
Loneliness sometimes (%)	30.8		22.3		19.6		24.2			
Loneliness occasionally (%)	17.1		10.4		10.8		15.2			
Loneliness most or all of the time (%)	8.5		20.1		12.5		12.2			
Work outside home (% yes)	51.29		10.5		1.8		60.6		89.64 (0.00)	0 & 3 > 1 & 2
Live with children (% yes)	41.1		17.9		7.2		24.3		25.32 (0.00)	0 > 1, 2 & 3
Care for other relatives (% yes)	35.1		11.9		3.6		9.1		34.87 (0.00)	0 > 1, 2 & 3
Hours of care	11.1	8.46	16.1	7.50	16.59	7.44	8.68	9.16	12.04	0 & 3 < 1 & 2
Frequency of disruptive behaviors	37.21	13.98	32.31	12.23	32.89	11.42	31.93	11.17	2.78 (0.03)	0 > 1, 2 & 3
Functional capacity of person cared for	61.96	27.77	71.65	26.17	7663	23.82	65.61	31.07	3.52 (0.01)	0 < 1 & 2
Family obligations	6.39	3.51	10.00	4.12	9.82	4.55	8.60	3.52	13.02 (0.00)	0 < 1, 2 & 3
Dysfunctional thoughts	19.03	11.12	34.78	15.47	37.98	12.18	25.06	12.29	27.94 (0.00)	1 & 2 > 0 & 3
Requesting help	2.95	0.71	1.83	0.97	2.05	1.02	2.18	0.91	2.52 (0.04)	0 > 1, 2 & 3
Receiving help	2.83	1.17	2.85	1.39	2.98	1.31	2.93	1.15	2.08 (0.08)	n.s
Social support	11.52	3.68	11.13	3.72	10.77	4.57	8.72	3.95	3.34 (0.01)	3 < 0, 1 & 2
Leisure	5.71	2.78	5.43	2.70	6.48	2.80	6.12	2.67	1.26 (0.29)	n.s

Note: SD = Standard Deviation; n.s = non-significant differences.

**Table 2:** Standardized and unstandardized regression weights by kinship.

			**Daughters**		
			**Unstandardized Estimates**	**S.E.**	**C.R.**
Family obligations	→	Dysfunctional thoughts	1.942 **	0.233	8.339
Dysfunctional thoughts	→	Requesting help	−0.013 *	0.006	−2.269
Requesting help	→	Receiving help	0.614 **	0.147	4.183
Receiving help	→	Social support	0.855 **	0.284	3.008
Dysfunctional thoughts	→	Leisure	−0.060 **	0.021	−2.867
Social support	→	Leisure	0.282 **	0.063	4.492
Leisure	→	Feeling of loneliness	−0.101 **	0.030	−3.358
Social support	→	Feeling of loneliness	−0.089 **	0.023	−3.911
			**Sons**		
			**Unstandardized Estimates**	**S.E.**	**C.R.**
Family obligations	→	Dysfunctional thoughts	1.802 **	0.523	3.442
Dysfunctional thoughts	→	Requesting help	0.018	0.013	1.376
Requesting help	→	Receiving help	0.744 **	0.187	3.984
Receiving help	→	Social support	1.093 ^†^	0.572	1.910
Dysfunctional thoughts	→	Leisure	−0.023	0.035	−0.665
Social support	→	Leisure	0.251 *	0.109	2.307
Leisure	→	Feeling of loneliness	−0.214 **	0.066	−3.256
Social support	→	Feeling of loneliness	0.026	0.044	0.587
			**Wives**		
			**Unstandardized Estimates**	**S.E.**	**C.R.**
Family obligations	→	Dysfunctional thoughts	1.992 **	0.392	5.076
Dysfunctional thoughts	→	Requesting help	−0.020 **	0.007	−2.722
Requesting help	→	Receiving help	0.842 **	0.147	5.710
Receiving help	→	Social support	0.580 ^†^	0.331	1.751
Dysfunctional thoughts	→	Leisure	−0.065 **	0.020	−3.300
Social support	→	Leisure	0.093	0.082	1.141
Leisure	→	Feeling of loneliness	−0.069	0.054	−1.265
Social support	→	Feeling of loneliness	−0.015	0.039	−0.394
			**Husbands**		
			**Unstandardized Estimates**	**S.E.**	**C.R.**
Family obligations	→	Dysfunctional thoughts	1.117 **	0.327	3.413
Dysfunctional thoughts	→	Requesting help	−0.019	0.011	−1.759
Requesting help	→	Receiving help	0.863 **	0.132	6.536
Receiving help	→	Social support	1.194 **	0.448	2.663
Dysfunctional thoughts	→	Leisure	−0.050	0.029	−1.745
Social support	→	Leisure	0.166 *	0.077	2.170
Leisure	→	Feeling of loneliness	−0.119 *	0.050	−2.394
Social support	→	Feeling of loneliness	−0.042	0.030	−1.397

Note: ^†^ = *p* < 0.10; * = *p* < 0.05; ** = *p* < 0.01; S.E. = Standard error; C.R. = Critical Ratio.

## Data Availability

The study materials, analytic methods and data are available upon request from the corresponding author on reasonable request.

## Supplementary Materials

The following are available online at https://www.mdpi.com/article/10.3390/ijerph18094700/s1, Table S1: Correlation matrix for husbands, Table S2: Correlation matrix for wives, Table S3: Correlation matrix for daughters, Table S4: Correlation matrix for sons.
