# Supplementary material for: Sociocultural Influences on the Feeling of Loneliness of Family Caregivers of People with Dementia: The Role of Kinship
Source: Int J Environ Res Public Health. 2021 Apr 28;18(9):4700. doi: 10.3390/ijerph18094700 (PMC8125119; doi:10.3390/ijerph18094700)
Supplement: Supplementary file 1 [file ijerph-18-04700-s001.zip › ijerph-1188171-supplementary.pdf]

Correlation matrix for husbands

VARIABLES	1	2	3	4	5	6	7	8	9	10	11	12	13
**1 .Age**													
**2. Feeling of loneliness**	−0.03												
**3. Work outside home**	0.26*	−0.10											
**4. Live with children**	−0.18	−0.01	−0.04										
**5. Care for others**	−0.27*	0.13	−0.03	−0.05									
**6. Frequency disruptive behaviors**	−0.14	0.35**	−0.11	0.04	−0.07								
**7. Functional capacity**	−0.23	−0.09	−0.15	0.06	0.01	−0.08							
**8. Hours of care**	−0.01	−0.09	−0.13	0.15	−0.12	0.15	0.20						
**9.Family obligations**	0.23	0.02	−0.20	0.04	−0.18	0.13	0.10	0.09					
**10.Dysfunctional Thoughts**	0.37**	−0.11	0.16	0.12	−0.03	−0.10	−0.07	0.31*	0.42**				
**11. Requesting help**	0.01	−0.07	0.13	0.19	−0.01	−0.11	−0.12	0.06	−0.34*	−0.23			
**12. Receiving help**	−0.10	−0.17	0.00	0.23	0.00	−0.26	−0.05	−0.02	−0.21	−0.18	0.67**		
**13. Social Support**	0.01	−0.27*	−0.02	0.20	−0.27	−0.16	−0.08	0.07	−0.23	−0.14	0.36**	0.35*	
**14. Leisure**	−0.37**	−0.36**	−0.17	−0.02	0.07	−0.21	0.21	−0.17	−0.04	−0.26	0.07	0.11	0.30*

* = p < .05; ** = p < .01

Correlation matrix for wives

VARIABLES	1	2	3	4	5	6	7	8	9	10	11	12	13
**1. Age**													
**2. Feeling of loneliness**	0.06												
**3. Work outside home**	−0.24*	−0.06											
**4. Live with children**	−0.25*	−0.06	−0.03										
**5. Care for others**	−0.15	−0.16	0.03	0.07									
**6. Frequency disruptive behaviors**	−0.01	−0.08	−0.19	−0.17	−0.11								
**7. Functional capacity**	−0.16	−0.13	−0.10	−0.12	0.19	0.14							
**8. Hours of care**	0.08	−0.05	−0.04	−0.08	−0.19	0.14	−0.03						
**9.Family obligations**	0.48**	−0.01	−0.16	−0.12	−0.09	−0.06	−0.04	0.10					
**10.Dysfunctional Thoughts**	0.47**	0.13	−0.19	−0.07	−0.04	−0.01	−0.23	0.02	0.53**				
**11. Requesting help**	0.01	−0.15	0.01	0.12	0.16	−0.14	0.02	−0.01	−0.23	−0.32**			
**12. Receiving help**	0.15	−0.26*	−0.04	0.02	−0.07	−0.02	−0.17	0.05	−0.01	−0.15	0.59**		
**13. Social Support**	−0.07	−0.07	0.05	−0.15	−0.07	−0.05	0.15	−0.20	−0.07	−0.09	0.15	0.22	
**14. Leisure**	−0.33**	−0.16	0.13	−0.02	−0.01	0.09	0.44*	0.22	−0.25*	−0.38**	0.25*	0.03	0.16

* = p < .05; ** = p < .01

Correlation matrix for daughters

VARIABLES	1	2	3	4	5	6	7	8	9	10	11	12	13
1 .Age													
2. Feeling of loneliness	−0.04												
3. Work outside home	−0.30**	−0.13											
4. Live with children	−0.23*	0.07	0.00										
5. Care for others	0.00	−0.21*	−0.14	0.01									
6. Frequency disruptive behaviors	−0.23*	0.16	0.19*	0.01	0.15								
7. Functional capacity	−0.24**	−0.02	−0.04	0.127	−0.09	0.16							
8. Hours of care	0.11	0.11	−0.25**	−0.08	−0.10	−0.07	−0.08						
9.Family obligations	0.08	0.02	−0.07	0.00	0.01	0.01	0.01	−0.01					
10.Dysfunctional Thoughts	0.27**	0.25**	−0.13	−0.02	−0.14	−0.13	−0.05	0.07	0.61**				
11. Requesting help	−0.19*	−0.11	0.02	0.09	0.07	−0.02	−0.00	−0.05	−0.14	−0.21*			
12. Receiving help	0.07	−0.31*	0.22*	−0.01	−0.02	−0.13	−0.09	−0.07	−0.04	−0.05	0.36*		
13. Social Support	−0.04	−0.45**	0.10	−0.03	−0.14	−0.28**	0.12	−0.03	0.12	−0.06	0.14	0.27**	
14. Leisure	−0.04	−0.42**	0.05	0.02	−0.05	−0.14	0.09	−0.05	−0.17	−0.26**	0.10	0.23*	0.39**

* = p < .05; ** = p < .01

Correlation matrix for sons

VARIABLES	1	2	3	4	5	6	7	8	9	10	11	12	13
**1 .Age**													
**2. Feeling of loneliness**	−0.10												
**3. Work outside home**	−0.50*	0.17											
**4. Live with children**	−0.06	−0.09	0.02										
**5. Care for others**	0.17	0.03	0.26	0.31+									
**6. Frequency disruptive behaviors**	−0.15	0.48	0.07	0.06	0.03								
**7. Functional capacity**	−0.17	−0.22	0.18	0.08	−0.02	0.00							
**8. Hours of care**	0.22	−0.14	−0.25	−0.14	0.09	0.02	0.08						
**9.Family obligations**	0.17	0.14	−0.13	−0.49**	−0.20	−0.04	0.08	0.12					
**10.Dysfunctional Thoughts**	0.29	−0.29	−0.37*	−0.27	0.04	−0.22	0.11	0.44**	0.51**				
**11. Requesting help**	−0.22	−0.21	−0.04	−0.04	−0.18	−0.17	0.42*	0.17	0.14	0.24			
**12. Receiving help**	−0.19	−0.30*	0.213	−0.11	−0.10	−0.32+	0.36*	0.04	0.08	0.19	0.58**		
**13. Social Support**	−0.20	−0.11	0.04	0.02	−0.19	−0.13	0.12	−0.20	0.15	−0.12	0.49**	0.30+	
**14. Leisure**	0.08	−0.50**	0.01	0.24	−0.13	−0.06	0.28	−0.18	−0.09	−0.15	0.19	0.23	0.38**

+ = p. < .10; * = p < .05; ** = p < .01
